# Supplementary material for: Systematic discovery of gene-environment interactions underlying the human plasma proteome in UK Biobank
Source: Nat Commun. 2024 Aug 26;15:7346. doi: 10.1038/s41467-024-51744-5 (PMC11347662; doi:10.1038/s41467-024-51744-5)
Supplement: Supplementary file 1 — Supplementary Information [file 41467_2024_51744_MOESM1_ESM.pdf]

Systematic discovery of gene-environment interactions underlying the human plasma proteome in UK Biobank

Robert F. Hillary, Danni A. Gadd, Zhana Kuncheva, Tasos Mangelis, Tinchi Lin, Kyle Ferber, Helen McLaughlin, Heiko Runz, Biogen Biobank Team, Riccardo E. Marioni, Christopher N. Foley, Benjamin B. Sun

Consortium member list:

Biogen Biobank team

Ellen Tsai, Chia-Yen Chen, Ketian Yu, Yunfeng Huang, Denis Baird, Danai Chasioti, Cynthia Gubbels, Stephanie Loomis, Eric Marshall, Adele Mitchell, Coro Paisán-Ruiz, Tinchi Lin, Kyle Ferber, Helen McLaughlin, Heiko Runz and Benjamin Sun.

Supplementary Note 1.

There were 13 instances in which we identified a gene-environment interaction that explained why variance QTL (vQTL) sites lacked genetic main effects on protein levels. These are highlighted in **Supplementary Data 16**. One example is detailed in full in the main text (for monocyte counts and FLT3LG levels). The remaining 12 examples are detailed in this Supplementary Note. Of note, the cut-off to be deemed a main effect QTL was a Bonferroni-corrected threshold of $p<3.4\times10^{-11}$. Some sites had main effect *p*-values that were slightly above this threshold and had suggestive main effects, whereas others have almost null main effect associations. The examples are arranged by exposure name in alphabetical order and then by protein name where exposures have >1 protein, also listed in alphabetical order.

1. CD163 and Alkaline Phosphatase Levels

CD163 or Cluster of Differentiation 163 is a scavenger receptor for the hemoglobin-haptoglobin complex and is a cell-surface marker for monocyte lineage cells. Shedding of CD163 to circulating CD163 occurs in multiple disease states including liver disease. High levels of alkaline phosphatase (data-field: 30610) are also indicative of comprised liver function, in particular hepatobiliary injuries[1,2]. This is one potential explanation for the observed positive correlations between CD163 and alkaline phosphatase in **Fig. 1**. **Fig. 1** shows that there is an apparent main effect of rs167537 genotype on CD163 levels; however, this failed to survive Bonferroni correction (p=1.38x10^{-7}). The effect is strongest in the highest tertile of alkaline phosphatase levels, but this is attenuated by weak negative associations in the other tertiles. This is not likely to represent a strong preclusion of genetic main effects by a GEI but is included here given the necessity of electing a consistent *p*-value threshold throughout the analyses.

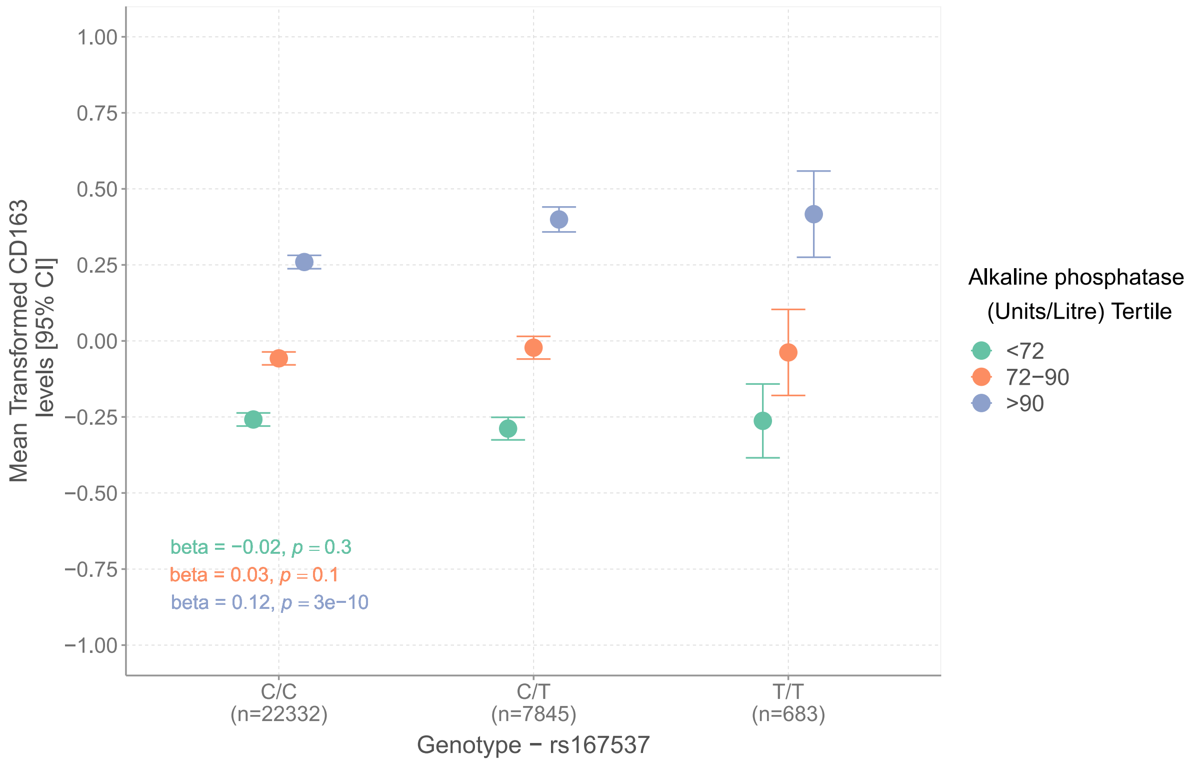

Fig. 1. GEI involving CD163 and alkaline phosphatase levels. CI, confidence interval.

2. ITGB7 and Alkaline Phosphatase Levels

The *trans* variant rs5409 annotated to *CLDN7* (Claudin 7) showed a weak main effect on ITGB7 (Integrin beta-7) levels (p=8.65x10^{-3}, **Fig. 2**). Alkaline phosphatase levels stratified individuals in the study population such that those in the highest and lowest tertiles showed opposing genetic effects on ITGB7 levels. The relationships between CLDN7 and ITGB7 remain unclear, and the relevance of ITGB7 to alkaline phosphatase levels also remains undocumented in the literature.

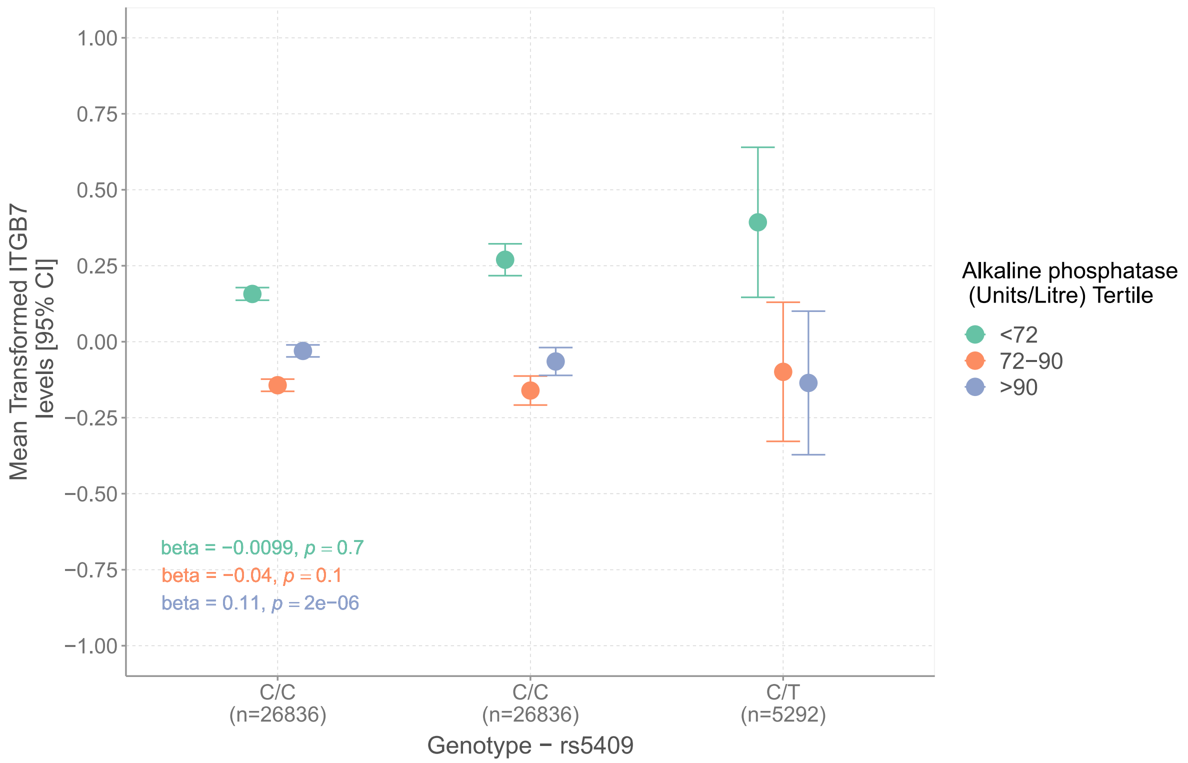

Fig. 2. GEI involving ITGB7 and alkaline phosphatase levels. CI, confidence interval.

3. BCAM and Apolipoprotein B Levels

Similar to the example for CD163 levels in **Fig. 1**, the following example for BCAM levels also points to a genetic main effect that failed to survive Bonferroni correction (p=5.28x10^{-8}, **Fig. 3**). Here, the *cis* variant rs111371860 was associated with BCAM levels, but was annotated to the nearby *NECTIN2* gene. *NECTIN2* encodes for Poliovirus receptor-related 2, which has been causally linked to increasing the production of low-density lipoprotein cholesterol levels[3,4]. Apolipoprotein B (data-field: 30640) is a major component of low-density lipoprotein cholesterol particles. Therefore, it is plausible that the genetic effects of *NECTIN2* on circulating BCAM levels are linked to Apolipoprotein B profiles. The highest and lowest tertiles showed genetic main effects of opposing directions, which resulted in an attenuation of the observed additive effect on BCAM protein levels.

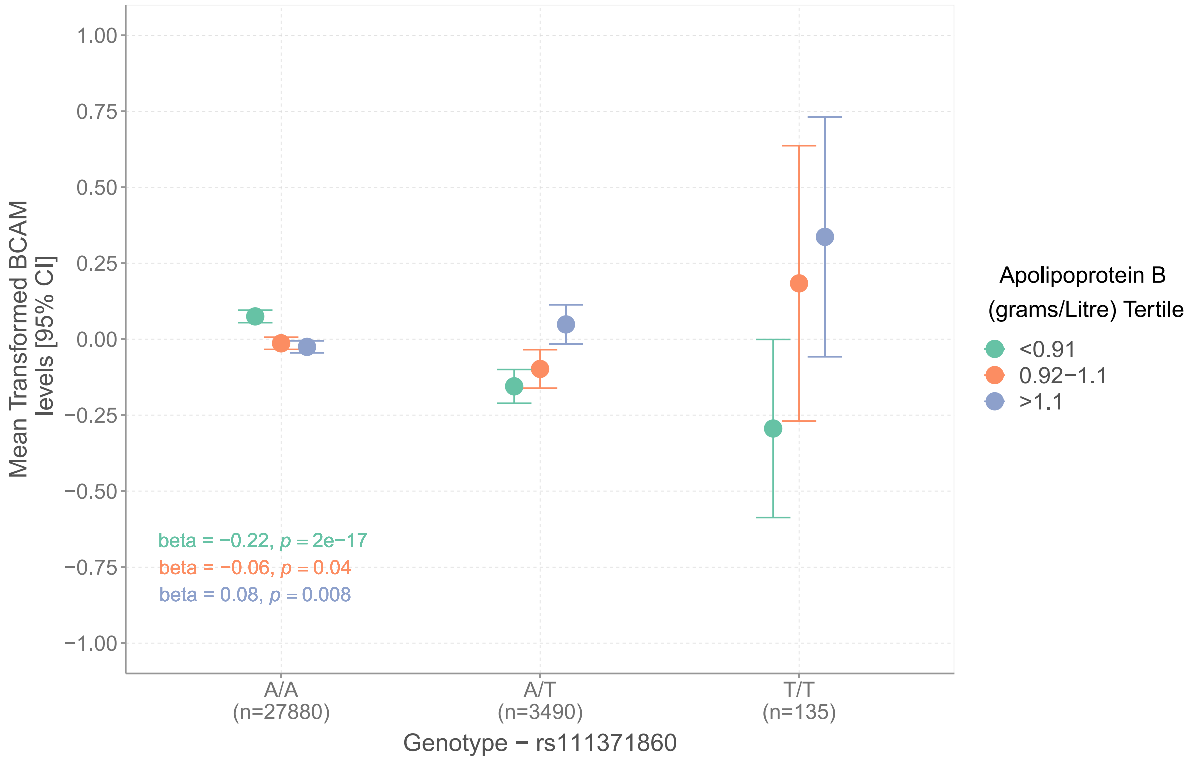

Fig. 3. GEI involving BCAM and apolipoprotein B levels. CI, confidence interval.

4. PLA2G7 and Apolipoprotein B Levels

PLA2G7 or Lipoprotein-associated phospholipase A2 travels with low-density lipoprotein cholesterol particles in the blood[5]. Therefore, it is unsurprising to observe such a strong correlation between apolipoprotein B levels and PLA2G7 levels (**Fig. 4**). There is a striking difference in the effect of rs75627662 genotype on PLA2G7 levels in those within the highest and lowest tertiles of apolipoprotein B levels, leading to a weak genetic main effect observation (p=1.55x10^{-3}).

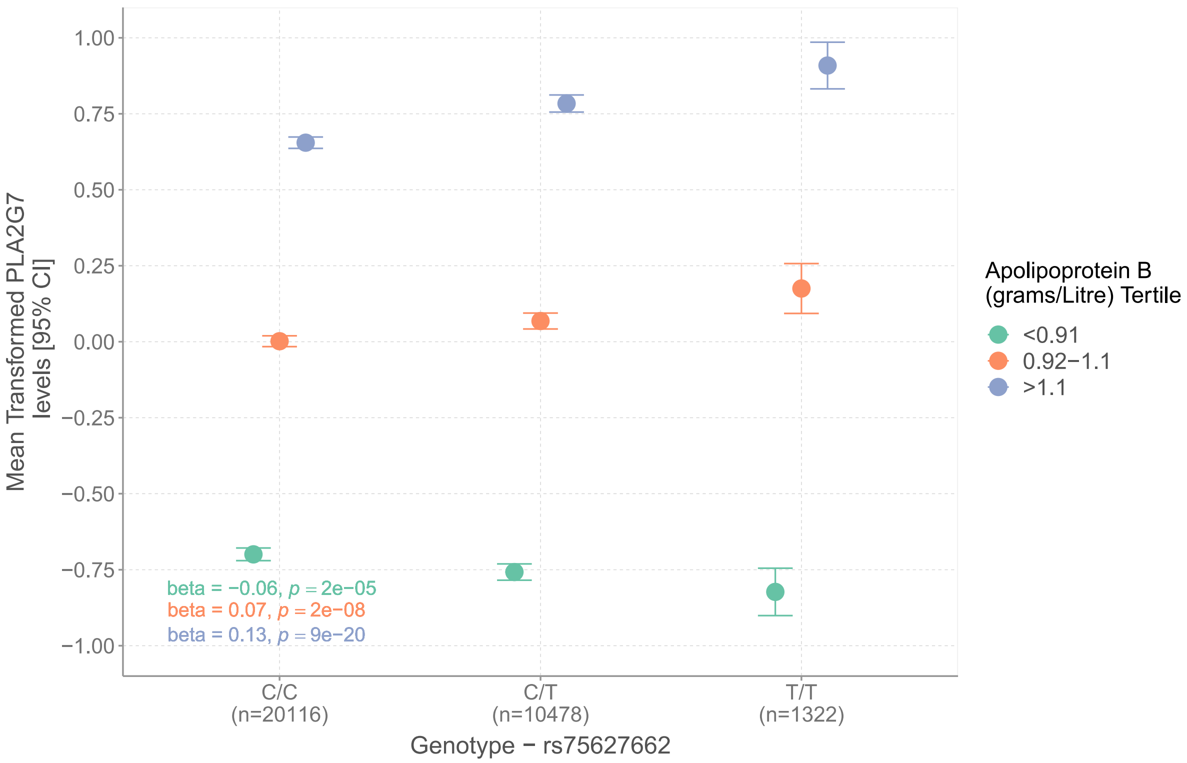

Fig. 4. GEI involving PLA2G7 and apolipoprotein B levels. CI, confidence interval.

5. ACY1 and Aspartate Aminotransferase Levels

Fig. 5 shows that ACY1 levels are correlated with aspartate aminotransferase levels across genotype groups at rs1883349, which was annotated to *SAMM50*. Polymorphisms within *SAMM50* have been linked to non-alcoholic fatty liver disease[6]. Similarly, aspartate aminotransferase levels (data-field: 30650) are elevated in liver disease[7]. ACY1 is therefore captured within this relationship between *SAMM50* and aspartate aminotransferase, such that a positive correlation between ACY1 and aspartate aminotransferase levels was observed in our study population. There was an apparent genetic main effect of rs1883349 on ACY1 levels that did not survive Bonferroni correction (p=1.45x10^{-10}). The mild negative effect of rs1883349 on ACY1 levels in those in the lower tertiles of aspartate aminotransferase levels attenuated the positive effect in the highest tertile.

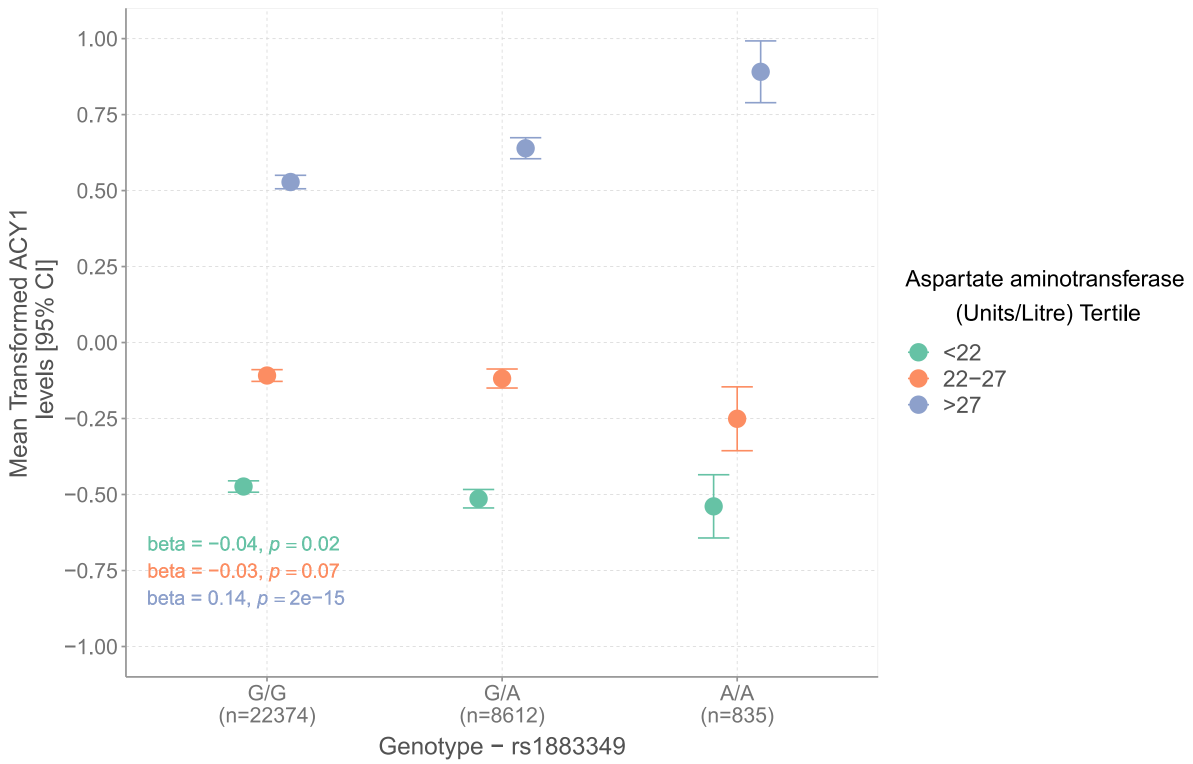

Fig. 5. GEI involving ACY1 and aspartate aminotransferase levels. CI, confidence interval.

6. CKMT1B and Aspartate Aminotransferase Levels

The variant rs1794519 was annotated to the complex *HLA* locus and associated with the variance of CKMT1B levels. There was no genetic main effect on protein levels (p=0.08, **Fig. 6**). There was a positive effect on CKMT1B levels in those within the highest tertile of aspartate aminotransferase levels, but there was no such association in the other tertiles. Separately, the increased variance in C allele carriers appears to result from a stronger positive correlation between CKMT1B and aminotransferase levels when compared to A allele carriers. CKMT1B reflects the ubiquitous mitochondrial creatine kinase protein. Creatine kinase and aspartate aminotransferase are both markers of muscle cell damage and exercise-associated strain[8], which may have prompted the positive correlation between these markers.

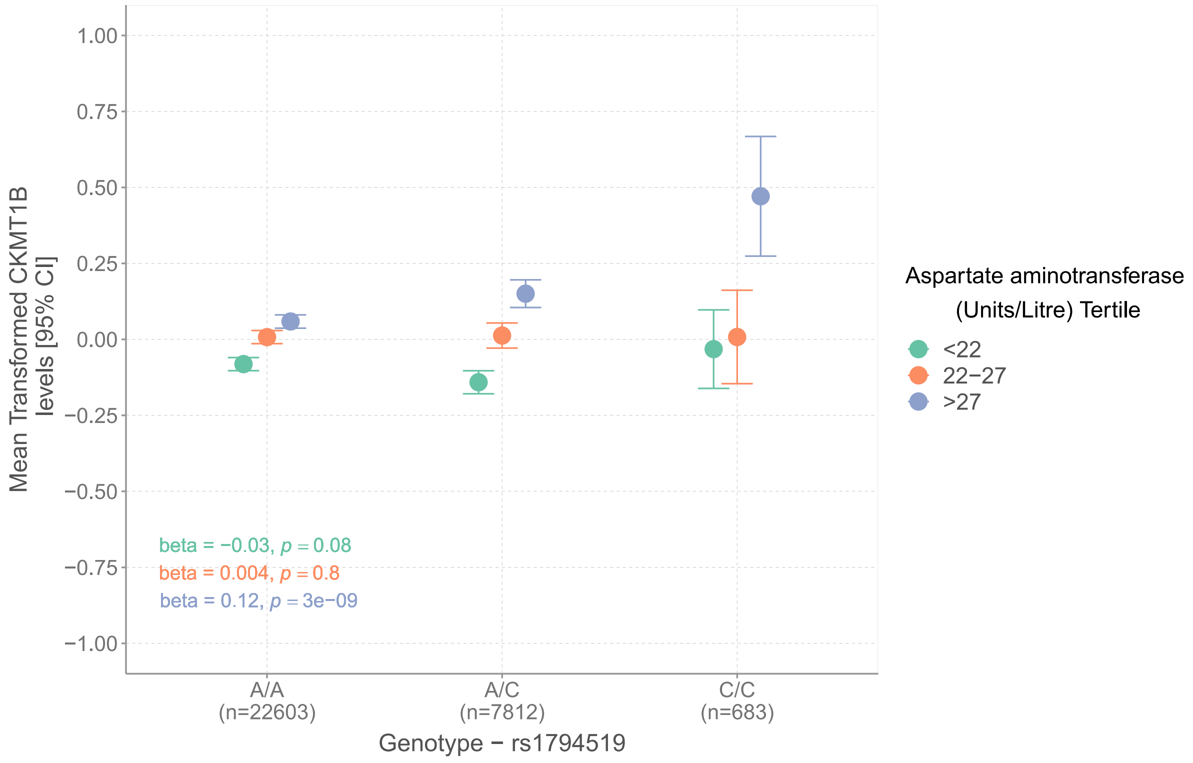

Fig. 6. GEI involving CKMT1B and aspartate aminotransferase levels. CI, confidence interval.

7. GUSB and Aspartate Aminotransferase Levels

The example for GUSB (p=5.47x10^{-10}, **Fig. 7**) largely mirrors that of **Fig. 5** for ACY1. It is also associated with a variant in *SAMM50* and likely implicates a similar relationship with aspartate aminotransferase levels.

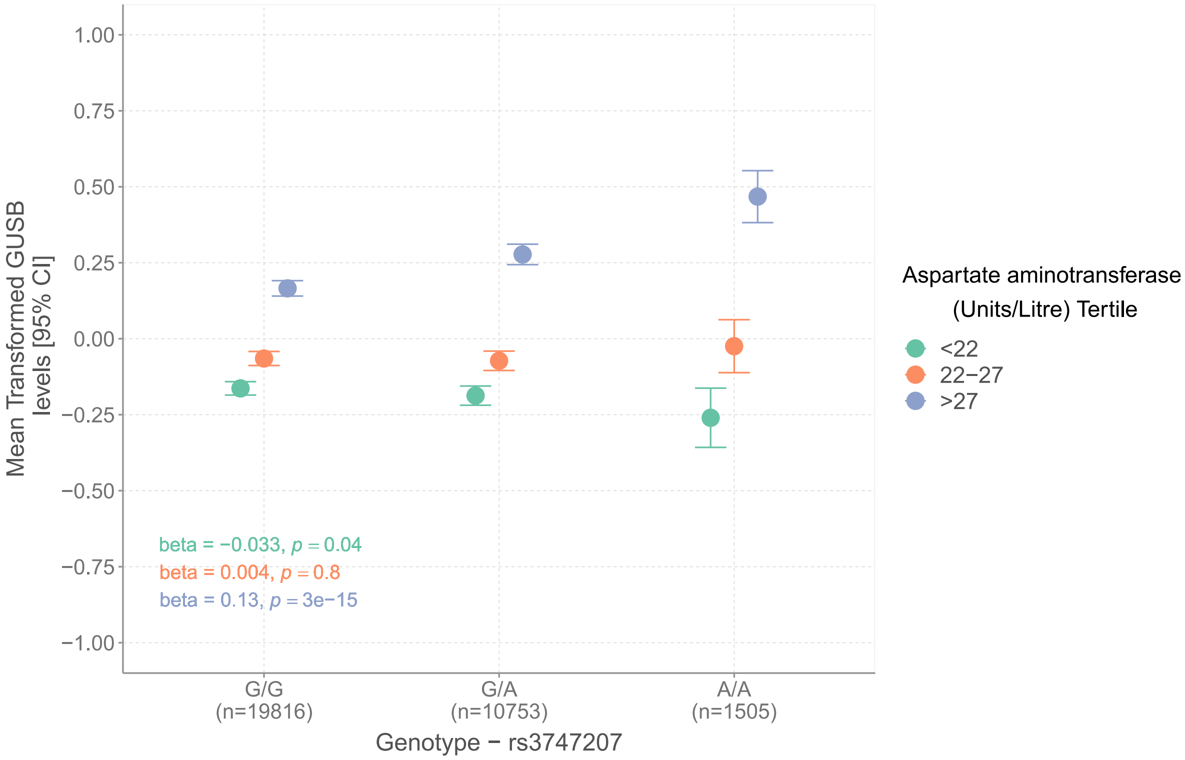

Fig. 7. GEI involving GUSB and aspartate aminotransferase levels. CI, confidence interval.

8. IGFBPL1 and Body Mass Index

The *cis* variant in *IGFBPL1* had a strong effect on the variance of its protein (p= 5.30x10^{-68}) but had a weaker effect on mean differences (p=3.79x10^{-9}). It also participated in a GEI association with measured body mass index (data-field 23104, **Fig. 8**). The genetic main effect on IGFBPL1 or Insulin-like growth factor-binding protein 1 levels appeared to be modulated by body mass index. Those in the highest tertile, approximately corresponding to obese participants, showed a negative association. Those in the overweight and normo-BMI categories exhibited a positive association, with a stronger association being observed in the normo-BMI category (i.e. lowest tertile), possibly indicating a dose-dependent relationship. The role of IGFBPL1 in adiposity is unclear; however it prolongs the half-life of IGFs, which show complex associations with adiposity and lipid profiles[9].

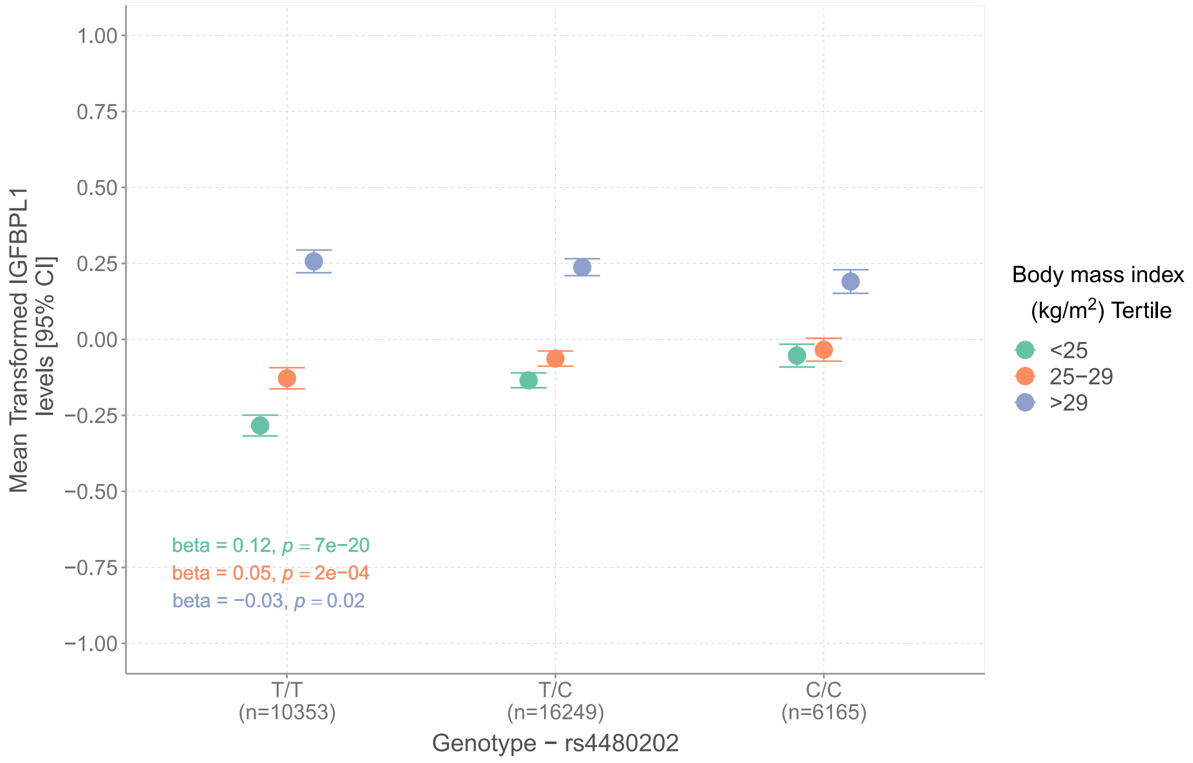

Fig. 8. GEI involving IGFBPL1 levels and body mass index. CI, confidence interval.

9. BST2 and Gamma Glutamyltransferase Levels

BST2 is also known as Bone marrow stromal antigen 2 or Tetherin. It forms an important part of pro-inflammatory pathways and is implicated in multiple immune cell types. BST2 showed positive correlations with gamma glutamyltransferase levels (data-field: 30730, **Fig. 9**). Gamma glutamlytransferase levels are upregulated in liver disease and in response to infections[10,11]. The observed positive correlation may arise from both proteins tracking poorer health in study participants. The opposing associations shown by the T-allele in the highest and lowest tertiles entirely precluded a genetic main effect observation (*p*=0.22).

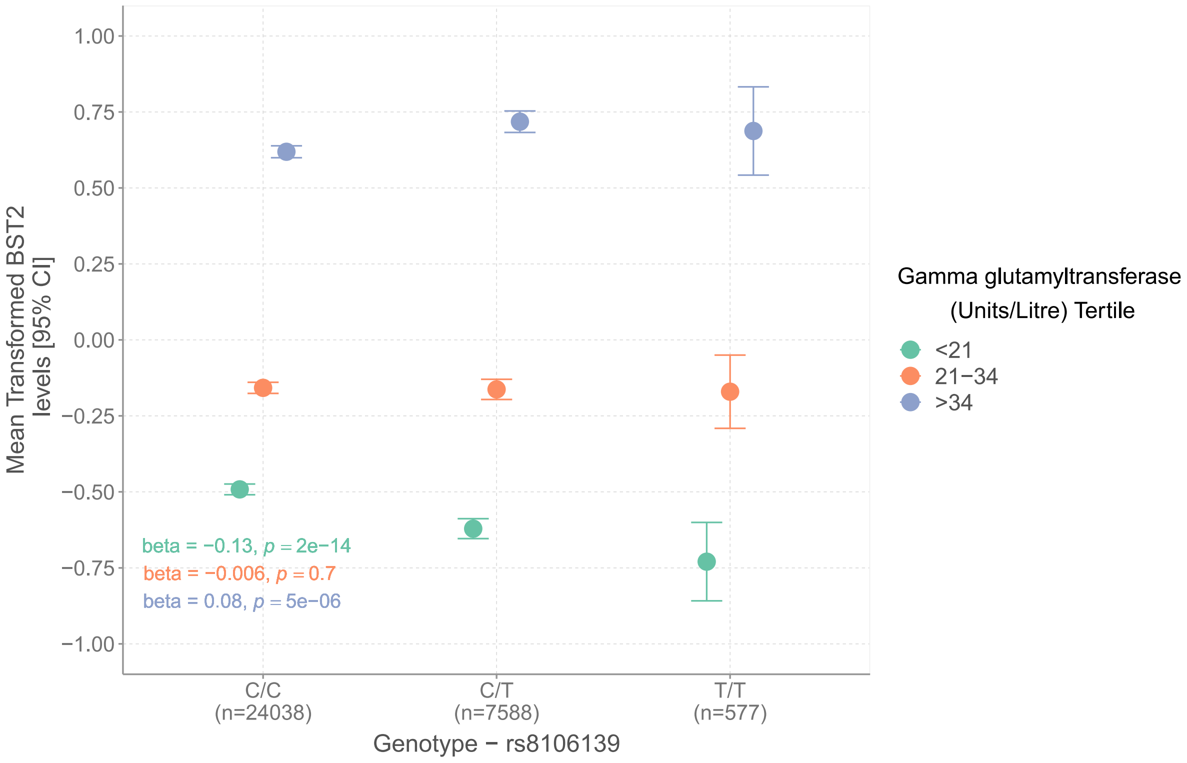

Fig. 9. GEI involving BST2 and gamma glutamyltransferase levels. CI, confidence interval.

10. GALNT2 and Leg Fat Percentage (Right Leg)

GALNT2 encodes for polypeptide N-acetyl-galactosaminyl-transferase 2, which has been routinely linked to lipid profiles and insulin sensitivity[12]. **Fig. 10** shows that GALNT2 levels exhibited a complex GEI association with right leg fat percentage (data-field: 23111), indicative of its involvement in regulating adiposity. The genetic main effect of rs34417180 on GALNT2 failed to survive Bonferroni correction due to stratification by leg fat percentage tertiles (p=1.90x10^{-7}).

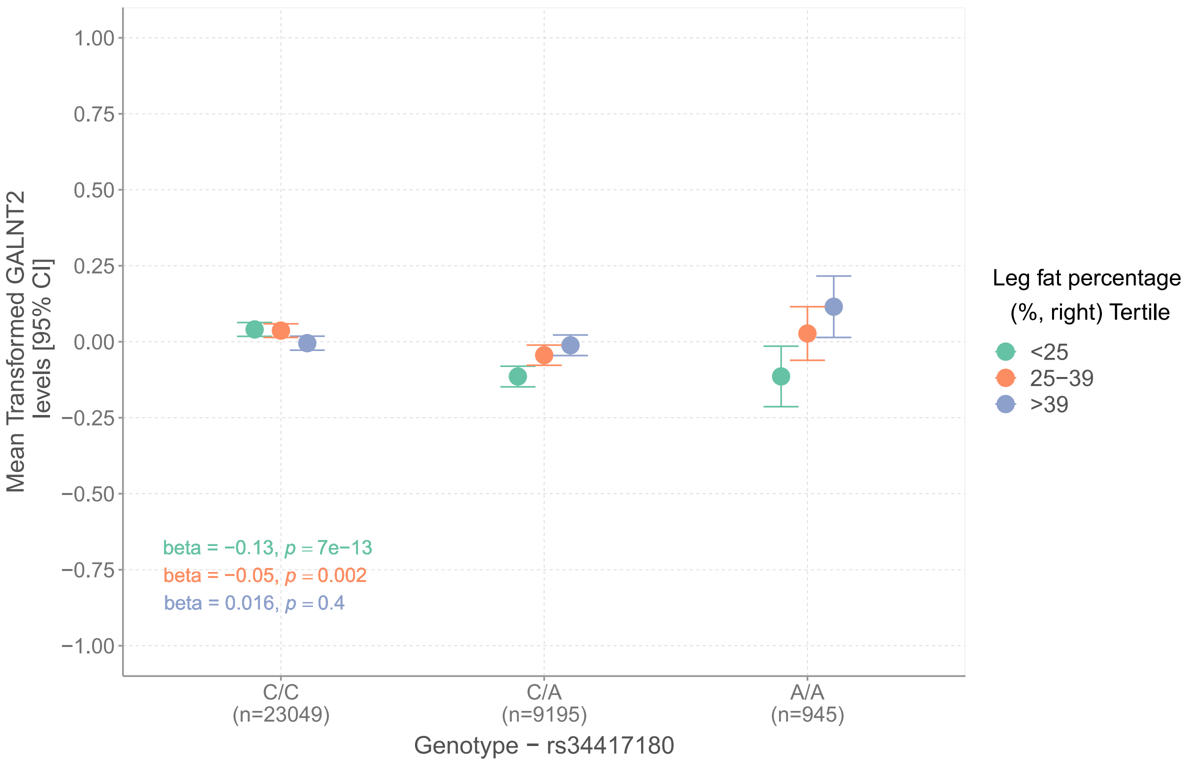

Fig. 10. GEI involving GALNT2 levels and right leg fat percentage. CI, confidence interval.

11. CPA2 and Pancreas Volume Levels

There was no clear genetic main effect of rs867983601 on CPA2 levels (p=1.40x10^{-4}, **Fig. 11**). CPA2 levels showed a GEI association with pancreas volume (data-field: 21087). However, there is no clear association between CPA2 levels and pancreas volume or function in the literature. Similarly, there was no clear correlation between CPA2 levels and pancreas volume in this study.

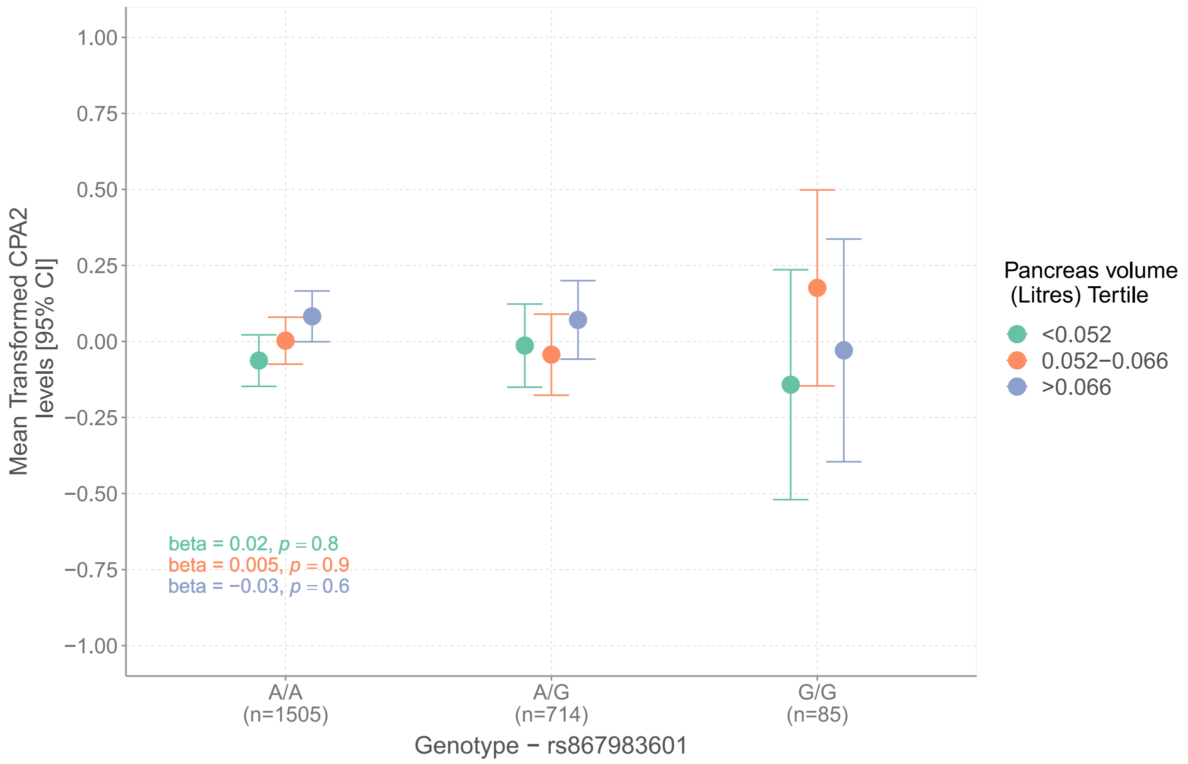

Fig. 11. GEI involving CPA2 levels and pancreas volume. CI, confidence interval.

12. CD160 and Polyunsaturated Fatty Acid Levels

The *trans* variant rs3096690, annotated to the *MHC* region, was associated with the variance of CD160 levels (**Fig. 12**). It did not however show a genetic main effect due to stratification by polyunsaturated fatty acid levels (data-field: 23446, p=0.02). Polyunsaturated fatty acid levels are negatively associated with dyslipidaemia and atherosclerosis[13]. CD160 levels are upregulated in the context of atherosclerosis[14]. This could point towards an explanation for the negative correlation observed between CD160 and polyunsaturated fatty acid levels in our study population.

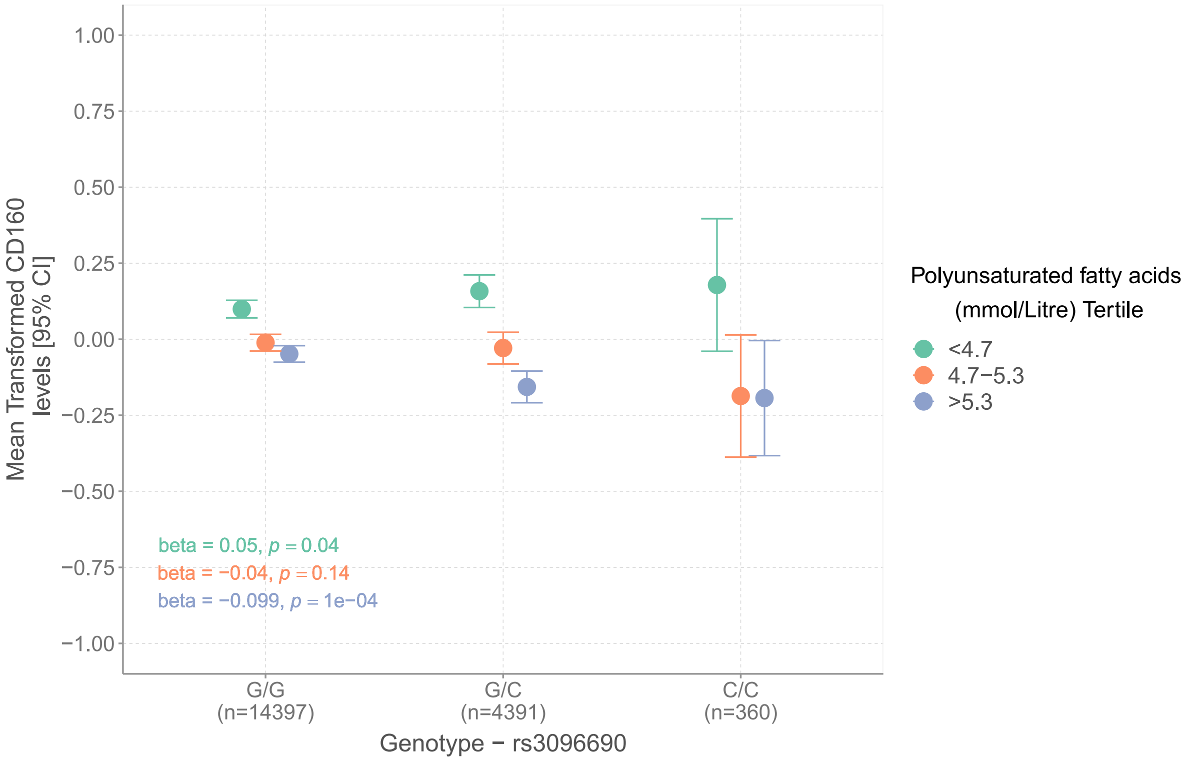

Fig. 12. GEI involving CD160 and polyunsaturated fatty acid levels. CI, confidence interval.

Supplementary Note 2.

Variance QTLs and gene-environment interactions withstand sensitivity analyses

As described in Zhang *et al.* (2016)[15] and Westerman *et al.* (2022)[16], the two-stage strategy employed in this study may have inflated type I error if an exposure associates with protein levels. In this case, test statistics from stage one and two might be correlated. Therefore, we repeated vQTL analyses in stage one but this time each protein that participated in a conditionally significant GEI in stage two was regressed onto the exposure(s) it associated with in stage two. Association statistics were then re-estimated for the 104 vQTL associations that were implicated in 130 conditional GEIs within stage two. Relative effect sizes were correlated 98.7% (95% CI=[98.0%, 99.1%]) with those in the main analytical strategy. Further, 101 vQTLs (97.1%) remained significant at $p<0.05$ in this exposure-adjusted sensitivity analyses and 92 associations (88.5%) remained at the Bonferroni-corrected threshold of $p<3.4\times10^{-11}$ (**Supplementary Data 20**).

Supplementary Methods

Tests for epistasis (gene-gene interactions)

We tested for associations of protein levels with an interaction term between each vQTL and any other SNP greater than 10Mb away on the same chromosome. Epistatic interactions can exist with SNPs on different chromosomes. However, we focused on *cis*-chromosomal interactions alone to enable computational feasibility. The –epistasis flag was used to perform pairwise tests for epistasis in PLINK v1.9. Each of the 677 vQTLs identified in our study were fitted as the first SNP in the interaction term, and we alternated the second SNP as any other SNP further than 10Mb away as outlined above. The protein that associated with the vQTL in discovery analyses was fitted as the dependent variable. A Bonferroni-corrected threshold was set at $p<5.0\times10^{-8}$ ($p<0.05$ adjusted for ~1 million tests).

Sensitivity analyses to capture phantom vQTLs (i.e. statistical artefacts)

It is possible that apparent differences in the variance of a trait are not due to a variance QTL but rather due to the variant being in LD with additional linked mean effect SNP(s). To address this concern, we first searched for mean effect or main effect QTLs for each analyte that harboured a significant vQTL association in our discovery analysis (n=575). The most significant main effect QTL association (at $p<3.4\times10^{-11}$) was extracted from the summary statistics of Sun *et al*[17]. We focused on one main effect QTL per protein following the findings of Ek *et al.* showing that the majority of phantom QTLs were revealed by one such variant[18]. Where possible, we regressed out all variance in analytes explained by the respective main effect SNP, in addition to all other covariates originally included in the discovery stage. Each vQTL association test (n=677) was repeated using the residuals from this regression. It is challenging to ascertain whether flagged associations are true phantom QTLs in the absence of whole genome sequencing data. Therefore, these associations can be thought of as statistical artefacts.

Sample selection

In the first wave of selection, 5,500 samples collected from participants during the baseline visit were pre-selected by the consortium members. Following this, 44,502 additional and representative samples were selected from baseline visits via a stratified selection on age, sex and UKB study centre. This strategy was employed to reduce the number of plates for the picking process. There were 50,002 samples following the first wave of selection. In the second wave of selection, 7,000 samples were selected. This included 1,020 samples selected by the consortium and 3,637 participants in the COVID-19 imaging study. Of these 3,637 participants, 1,270 samples from baseline, pre-COVID and post-COVID imaging visits were included where possible. An additional 2,343 baseline samples were included in the second stage and were selected randomly as in the first stage in order to optimise the inclusion of selection locations required for the COVID imaging and consortium selected samples. Consortium members chose samples that were enriched for specific diseases of interest. Day of the week of collection, deprivation index and participant ethnicity were representative of

the wider UK Biobank cohort. Full inclusion and eligibility criteria are detailed in: https://biobank.ndph.ox.ac.uk/showcase/showcase/docs/casecontrol_covidimaging.pdf.

Proteomic profiling and quality control

In the Proximity Extension Assay, blood samples are incubated in the presence of proximity antibody pairs linked to DNA reporter molecules. Upon binding of an antibody pair to their corresponding antigen, the respective DNA tails form an amplicon by proximity extension, which can be quantified by high-throughput real-time PCR. This method limits the reporting of cross-reactive events. Amplicons were combined across each of four distinct abundance groups resulting in one well of amplicons per sample. Four unique 96-index plates were added to every four sample plates followed by a second PCR reaction, which enabled all samples in a plate to be combined into one library per panel. Each library was bead-purified using a Bioanalyzer. Four sample plates from four identical panels were pooled, denatured and sequenced on individual lanes on the Novaseq600 using S4 flow cells v1.5 (35 cycles) and 384-samples and 384-assays measured. Counts of known sequences were translated in to Normalized Protein eXpression (NPX) values within Olink's MyData Cloud Software.

Raw data were generated for 54,219 individuals. Olink's inbuilt QC system includes three controls (incubation, extension and amplification controls) that are spiked into every sample and to each abundance block. Each sample also includes external controls in the twelfth column and a triplicate negative control is added to aid in calculating limits of detection. The proteins IL6, IL8 (CXCL8), and TNF were present across several panels and helped to assess consistency and correlations of protein measurements. Olink samples were divided into eight batches. Samples were randomised across 96-well plates. The first seven batches (labelled batch 0-6) included participants from UKB baseline. The eighth batch (labelled batch 7) contain samples from the baseline and the COVID imaging study. NPX values were calculated by subtracting the assay-specific median value of plate controls from the $\log_2$ ratio of counts of each assay-sample pair to the counts of the extension control. This was performed separately for baseline and COVID imaging samples. This provided plate normalized NPX values for both sets. For baseline samples, batch-specific median NPX values were added per assay in order to account for batch-to-batch variation. Data were then normalised within each of the batches. Adjustment factors were calculated using batch 1 as the reference batch and added to baseline-only batches. Adjustment factors for batch 7 were computed from the assay-specific median of the pairwise differences between bridge samples in batches 0-6 and batch 7. These adjustment factors were added to NPX values within batch 7.

Samples were removed if they were (i) Olink QC failures, (ii) outliers that had a standardised principal component (PC) 1 or 2 beyond 5 standard deviations from the mean (zero-standardised PCA), (iii) outliers with a median NPX greater than 5 standard deviations from the mean or an interquartile range (IQR) greater than 5 standard deviations from the mean IQR and (iv) flagged by QC or assay warnings. These criteria left measurements for 52,790

individuals for analyses. Baseline samples for these 52,790 individuals were taken forward for use in the present study.

vQTL association studies

vQTL effect sizes and their standard errors are not natively produced by Levene's test. First, a direction of effect is inferred by regressing absolute deviations of phenotype values from the median onto additively-coded genotype values (0, 1 or 2). Second, effect sizes and standard errors are back-transformed using the sign of the effect's direction and the test's *p*-value.

Phenotype preparation for gene-environment interaction association tests

Education (data-field: 6138) was binarised to having completed college/university education or not. Season of blood draw was included as a sole technical exposure due to its potential influence on inter-individual variation in protein measurements [19]. Season of blood draw was based on the blood collection date and time (data-field: 3166) and binarised to 'Summer/Autumn' (June to November) and 'Winter/Spring' (December to May).

Supplementary Figures.

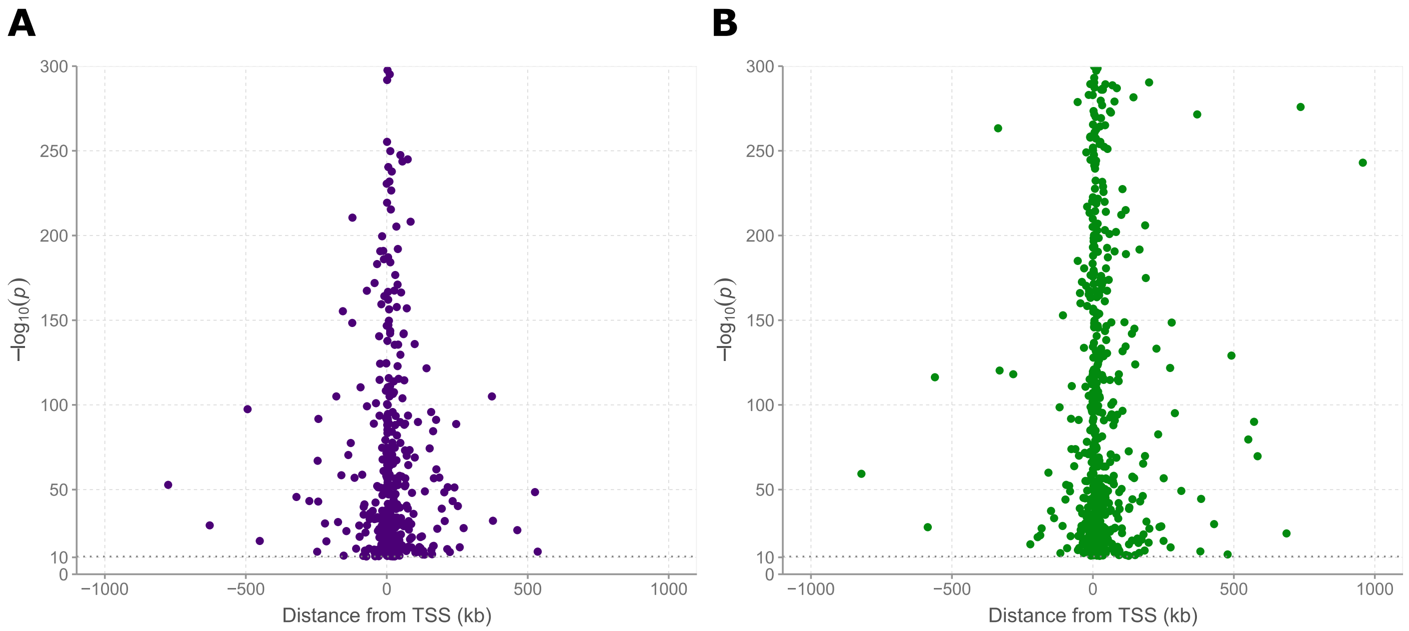

Supplementary Fig. 1. **Significance of QTL associations versus distance of variants from the gene transcription start site (TSS).** Closed circles show the -$\log_{10}(p)$ value for *cis* vQTL associations (**A**; indigo) and pQTL associations (**B**; emerald). The horizontal dotted line denotes the Bonferroni-corrected significance threshold used in vQTL and pQTL association analyses, which reflects $p<3.4\times10^{-11}$ or -$\log_{10}(p)$~10.5. Distances are capped at 1,000 kilobases (kb) or 1 Megabase for clarity. Kb, kilobase; pQTL, protein quantitative trait locus; TSS, transcription start site; vQTL, variance quantitative trait locus.

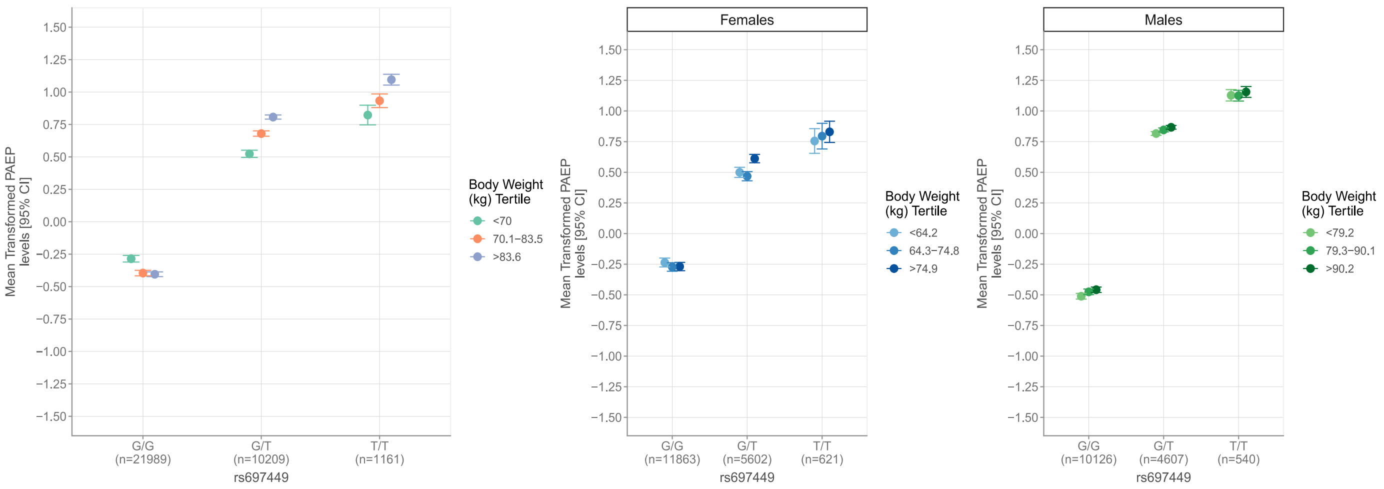

Supplementary Fig. 2. Relationship between glycodelin and body composition stratified by males and females. **(A)** Mean transformed glycodelin levels (PAEP, closed circles) with 95% confidence intervals (vertical bars) are shown according to tertiles of body weight (in kilograms, kg) and rs697449 genotype. **(B)** The plots are then stratified into males (green) and females (blue).

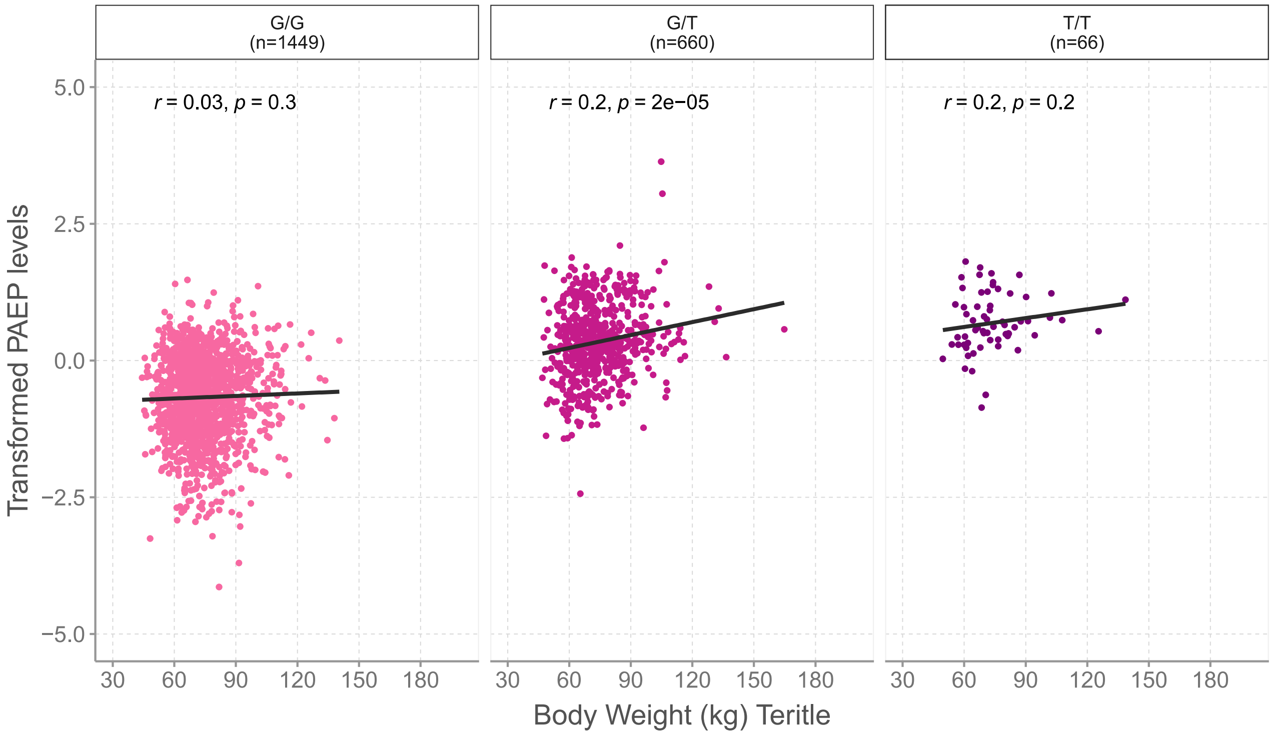

Supplementary Fig. 3. Relationship between glycodelin and body composition in females who self-reported having had hysterectomy. Correlations between PAEP levels and body weight are shown for participants who reported 'Not sure – had a hysterectomy' to the question 'Had menopause?' at the study baseline (Field: 2724). Correlations are stratified by rs697449 genotype.

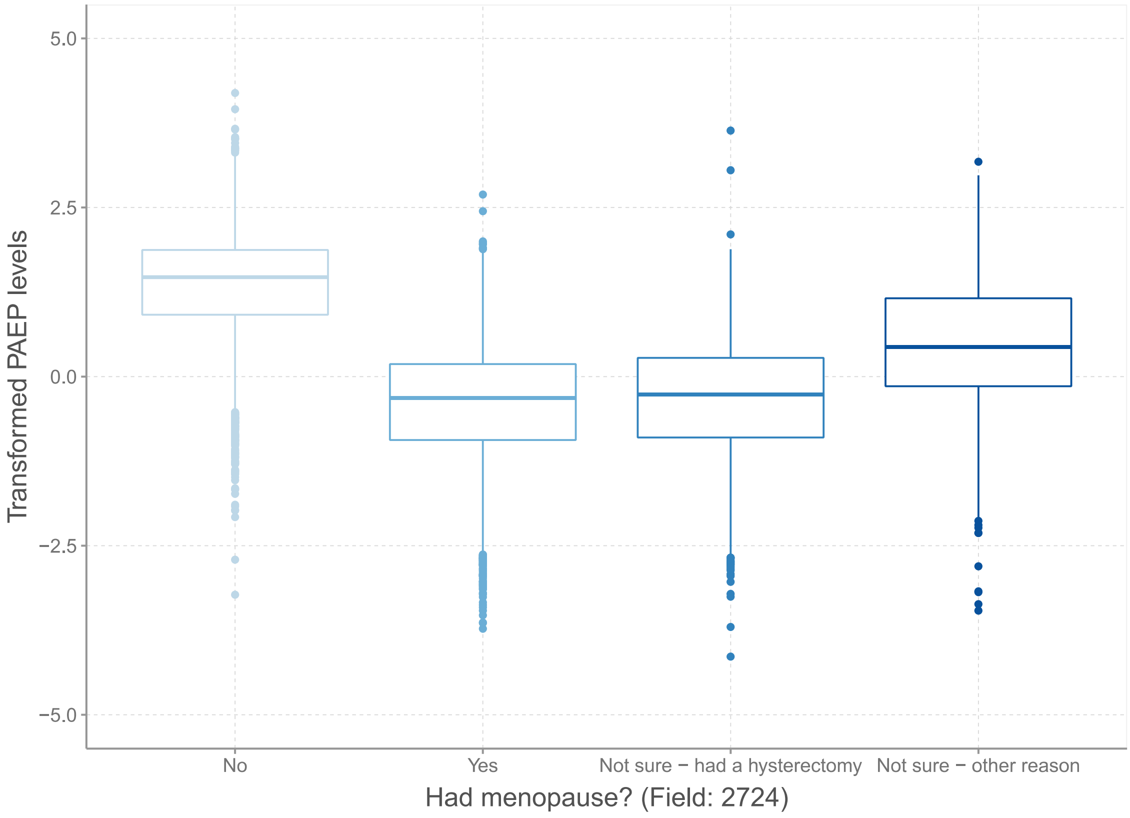

Supplementary Fig. 4. Relationship between PAEP levels and history of menopause. Boxplots of transformed PAEP levels according to responses to the question 'Had menopause?' at the study baseline (Field: 2724). Centre line of boxplot: median, bounds of box: first and third quartiles and tips of whiskers: minimum and maximum.

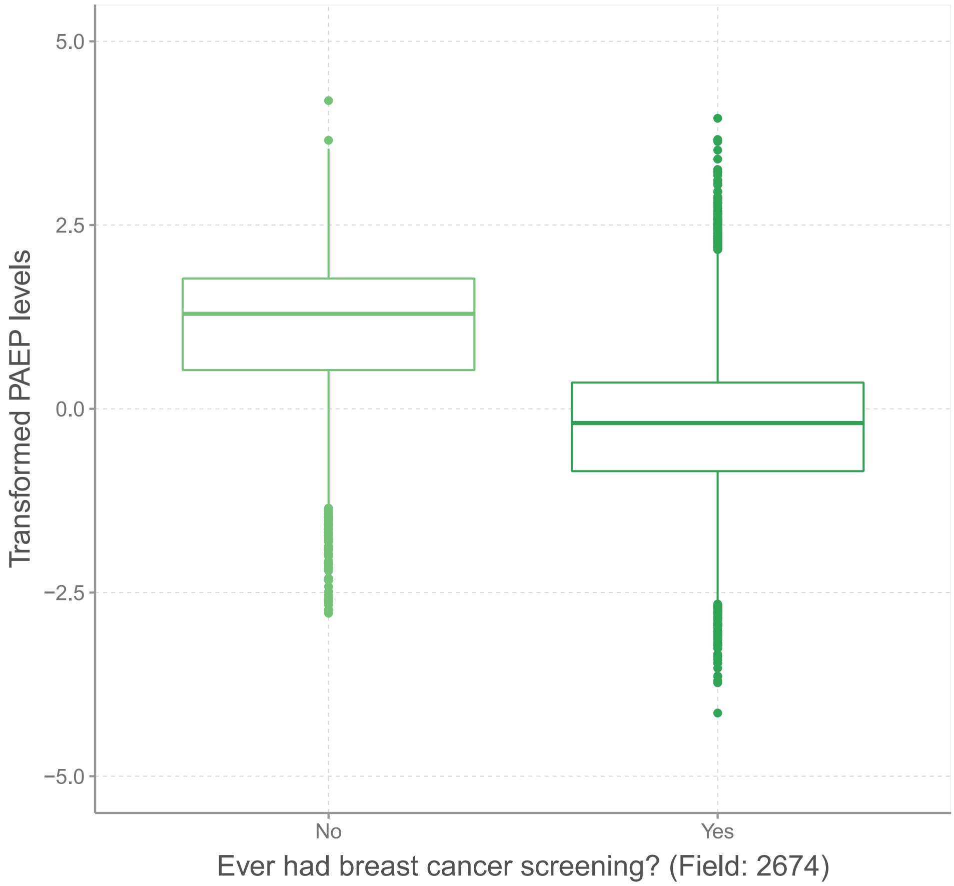

Supplementary Fig. 5. Relationship between PAEP levels and history of breast cancer screening. Boxplots of transformed PAEP levels according to responses to the question 'Ever had breast cancer screening / mammogram?' at the study baseline (Field: 2674). The mean ages for those who responded 'No' was 46.1 years (sd=4.5 years) and 'Yes' was 59.3 years (sd=6.4 years). Centre line of boxplot: median, bounds of box: first and third quartiles and tips of whiskers: minimum and maximum. sd, standard deviation.

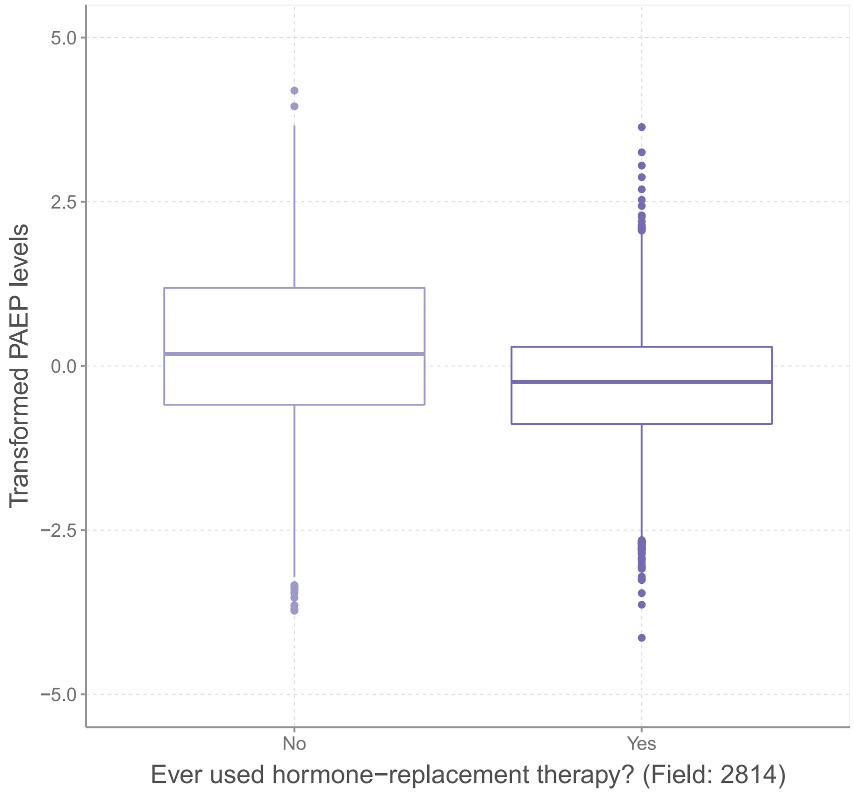

Supplementary Fig. 6. **Relationship between PAEP levels and history of hormone replacement therapy.** Boxplots of transformed PAEP levels according to responses to the question 'Ever used hormone-replacement therapy?' at the study baseline (Field: 2814). The mean ages for those who responded 'No' was 54.2 years (sd=8.2 years) and 'Yes' was 60.8 years (sd=5.5 years). Centre line of boxplot: median, bounds of box: first and third quartiles and tips of whiskers: minimum and maximum. sd, standard deviation.

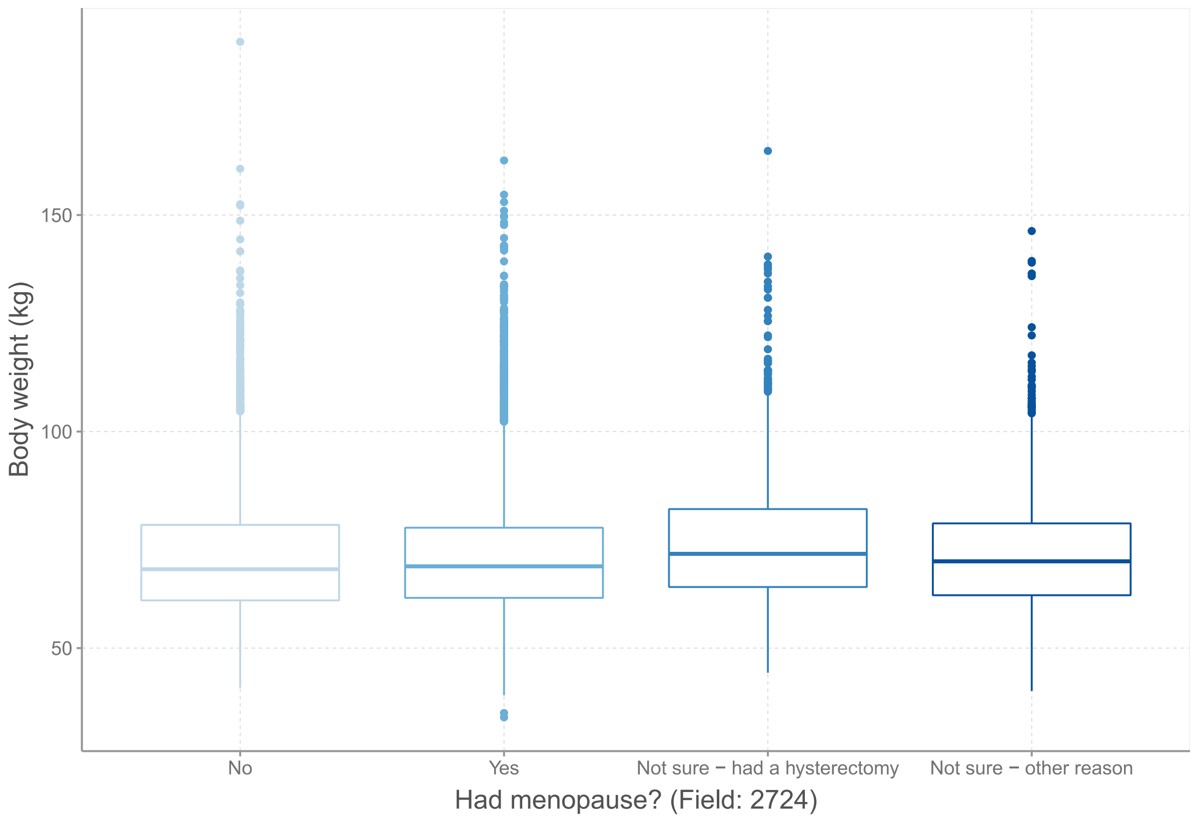

Supplementary Fig. 7. **Relationship between body weight and history of menopause.** Boxplots of body weight according to responses to the question 'Had menopause?' at the study baseline (Field: 2724). Centre line of boxplot: median, bounds of box: first and third quartiles and tips of whiskers: minimum and maximum.

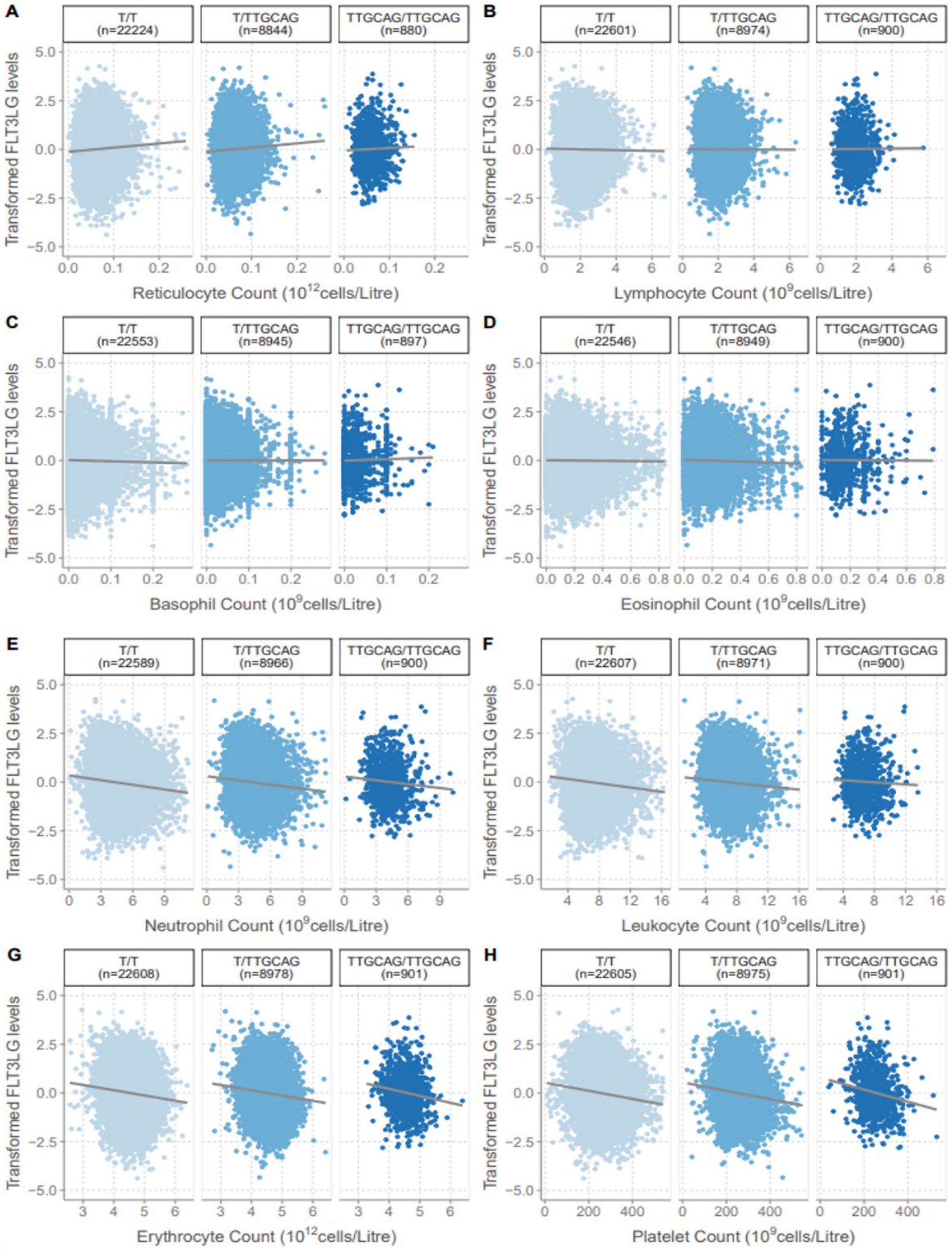

Supplementary Fig. 8. Correlation between FLT3LG levels and eight different blood cell types stratified by genotype at variance QTL for FLT3LG levels. (A) Reticulocyte counts. **(B)** Lymphocyte counts. **(C)** Basophil counts. **(D)** Eosinophil counts. **(E)** Neutrophil counts. **(F)** Leukocyte counts. **(G)** Erythrocyte counts. **(H)** Platelet counts. QTL, quantitative trait locus.

Supplementary References

1. Bossen, L. *et al.* Soluble CD163 and mannose receptor as markers of liver disease severity and prognosis in patients with primary biliary cholangitis. *Liver Int* **40**, 1408-1414 (2020).
2. Aulbach, A.D. & Amuzie, C.J. Chapter 17 - Biomarkers in Nonclinical Drug Development. in *A Comprehensive Guide to Toxicology in Nonclinical Drug Development (Second Edition)* (ed. Faqi, A.S.) 447-471 (Academic Press, Boston, 2017).
3. Rossignoli, A. *et al.* Poliovirus Receptor-Related 2: A Cholesterol-Responsive Gene Affecting Atherosclerosis Development by Modulating Leukocyte Migration. *Arterioscler Thromb Vasc Biol* **37**, 534-542 (2017).
4. van der Graaf, A. *et al.* Mendelian randomization while jointly modeling cis genetics identifies causal relationships between gene expression and lipids. *Nat Commun* **11**, 4930 (2020).
5. Tellis, C.C. & Tselepis, A.D. Pathophysiological role and clinical significance of lipoprotein-associated phospholipase A_2 (Lp-PLA_2) bound to LDL and HDL. *Curr Pharm Des* **20**, 6256-69 (2014).
6. Li, Z. *et al.* The role of SAMM50 in non-alcoholic fatty liver disease: from genetics to mechanisms. *FEBS Open Bio* **11**, 1893-1906 (2021).
7. Leoni, S. *et al.* Current guidelines for the management of non-alcoholic fatty liver disease: A systematic review with comparative analysis. *World J Gastroenterol* **24**, 3361-3373 (2018).
8. Brancaccio, P., Maffulli, N., Buonauro, R. & Limongelli, F.M. Serum enzyme monitoring in sports medicine. *Clin Sports Med* **27**, 1-18, vii (2008).
9. Sherlala, R.A. *et al.* Relationship Between Serum IGF-1 and BMI Differs by Age. *J Gerontol A Biol Sci Med Sci* **76**, 1303-1308 (2021).
10. Shao, T. *et al.* Gamma-Glutamyltransferase Elevation Is Frequent in Patients With COVID-19: A Clinical Epidemiologic Study. *Hepatol Commun* **4**, 1744-1750 (2020).
11. Koenig, G. & Seneff, S. Gamma-Glutamyltransferase: A Predictive Biomarker of Cellular Antioxidant Inadequacy and Disease Risk. *Dis Markers* **2015**, 818570 (2015).
12. Antonucci, A., Marucci, A., Trischitta, V. & Di Paola, R. Role of GALNT2 on Insulin Sensitivity, Lipid Metabolism and Fat Homeostasis. *Int J Mol Sci* **23**(2022).
13. Dessì, M. *et al.* Atherosclerosis, dyslipidemia, and inflammation: the significant role of polyunsaturated Fatty acids. *ISRN Inflamm* **2013**, 191823 (2013).
14. Zuo, J. *et al.* Increased CD160 expression on circulating natural killer cells in atherogenesis. *J Transl Med* **13**, 188 (2015).
15. Zhang, P., Lewinger, J.P., Conti, D., Morrison, J.L. & Gauderman, W.J. Detecting Gene-Environment Interactions for a Quantitative Trait in a Genome-Wide Association Study. *Genet Epidemiol* **40**, 394-403 (2016).
16. Westerman, K.E. *et al.* Variance-quantitative trait loci enable systematic discovery of gene-environment interactions for cardiometabolic serum biomarkers. *Nature Communications* **13**, 3993 (2022).
17. Sun, B.B. *et al.* Plasma proteomic associations with genetics and health in the UK Biobank. *Nature* **622**, 329-338 (2023).
18. Ek, W.E. *et al.* Genetic variants influencing phenotypic variance heterogeneity. *Hum Mol Genet* **27**, 799-810 (2018).
19. Enroth, S., Hallmans, G., Grankvist, K. & Gyllensten, U. Effects of Long-Term Storage Time and Original Sampling Month on Biobank Plasma Protein Concentrations. *EBioMedicine* **12**, 309-314 (2016).
