# Supplementary material for: Systematic discovery of gene-environment interactions underlying the human plasma proteome in UK Biobank
Source: Nat Commun. 2024 Aug 26;15:7346. doi: 10.1038/s41467-024-51744-5 (PMC11347662; doi:10.1038/s41467-024-51744-5)
Supplement: Supplementary file 3 — Description of Additional Supplementary Files [file 41467_2024_51744_MOESM3_ESM.pdf]

Description of Additional Supplementary Files

File Name: Supplementary Data 1
Description: Genomic inflation factors from all 1,472 genome-wide variance QTL association studies and LODs.

File Name: Supplementary Data 2
Description: Independent variance QTLs at $p<3.4\text{x}10^{-11}$ (LD-pruned, Bonferroni-corrected threshold).

File Name: Supplementary Data 3
Description: Annotation of vQTL variants using Open Targets platform.

File Name: Supplementary Data 4
Description: Variance QTL association statistics in replication set.

File Name: Supplementary Data 5
Description: Sensitivity analyses comparing association statistics in individuals of European and non-European ancestries within replication set.

File Name: Supplementary Data 6
Description: Look-up analyses for main effect QTLs in Sun *et al.* (2023).

File Name: Supplementary Data 7
Description: Sensitivity analyses to assess if vQTL genotypes associated with individuals falling above or below the limit of detections.

File Name: Supplementary Data 8
Description: Sensitivity analyses to assess if vQTL genotypes associated with missingness due to technical factors.

File Name: Supplementary Data 9
Description: Summary data for phenotypes used in gene-environment interaction or GEI tests.

File Name: Supplementary Data 10
Description: All significant GEI associations with variance QTLs at $p<1.4\text{x}10^{-7}$ (Bonferroni-corrected threshold).

File Name: Supplementary Data 11
Description: All significant GEI associations with main effect QTLs at $p<1.4\text{x}10^{-7}$ (Bonferroni-corrected threshold).

File Name: Supplementary Data 12
Description: Conditionally significant GEI associations in discovery set at $\text{p}<1.4\text{x}10^{-7}$ (Bonferroni-corrected threshold).

File Name: Supplementary Data 13
Description: Conditionally significant GEI associations in replication set.

File Name: Supplementary Data 14
Description: Direct associations between protein levels and exposures shown in **Fig. 4A**.

File Name: Supplementary Data 15
Description: Subset of conditional GEIs that involved phenotypes uniquely identified through GWAS Catalog look-up analyses.

File Name: Supplementary Data 16
Description: Direction of associations between genotype and protein levels across different strata of exposures from **Supplementary Data 12**.

File Name: Supplementary Data 17
Description: Assessment of epistasis - associations of protein levels with interactions between vQTLs and SNPs on the same chromosome.

File Name: Supplementary Data 18
Description: Assessment of phantom vQTLs - conditioning on most significant main effect SNPs on the same chromosome as vQTLs.

File Name: Supplementary Data 19
Description: Sensitivity variance QTL analyses using phenotype preparation method recommended by Wang *et al.* (2019).

File Name: Supplementary Data 20
Description: Sensitivity variance QTL analyses using protein levels that were regressed on partner phenotype(s) in conditional GEI associations.

File Name: Supplementary Data 21
Description: Summary data for demographic variables and covariates in UKB-PPP sample.

File Name: Supplementary Data 22
Description: Associations between proteins and covariates in the discovery set.

File Name: Supplementary Data 23
Description: Associations between proteins and covariates in the replication set.
