# Supplementary material for: Systematic discovery of gene-environment interactions underlying the human plasma proteome in UK Biobank
Source: Nat Commun. 2024 Aug 26;15:7346. doi: 10.1038/s41467-024-51744-5 (PMC11347662; doi:10.1038/s41467-024-51744-5)
Supplement: Supplementary file 27 — Reporting Summary [file 41467_2024_51744_MOESM27_ESM.pdf]

nature portfolio

Corresponding author(s): Riccardo E Marioni and Benjamin B Sun

Last updated by author(s): Jun 1, 2024

Reporting Summary

Nature Portfolio wishes to improve the reproducibility of the work that we publish. This form provides structure for consistency and transparency in reporting. For further information on Nature Portfolio policies, see our Editorial Policies and the Editorial Policy Checklist.

Statistics

For all statistical analyses, confirm that the following items are present in the figure legend, table legend, main text, or Methods section.

n/a	Confirmed	
☐	☒	The exact sample size (*n*) for each experimental group/condition, given as a discrete number and unit of measurement
☐	☒	A statement on whether measurements were taken from distinct samples or whether the same sample was measured repeatedly
☐	☒	The statistical test(s) used AND whether they are one- or two-sided<br>*Only common tests should be described solely by name; describe more complex techniques in the Methods section.*
☐	☒	A description of all covariates tested
☐	☒	A description of any assumptions or corrections, such as tests of normality and adjustment for multiple comparisons
☐	☒	A full description of the statistical parameters including central tendency (e.g. means) or other basic estimates (e.g. regression coefficient) AND variation (e.g. standard deviation) or associated estimates of uncertainty (e.g. confidence intervals)
☐	☒	For null hypothesis testing, the test statistic (e.g. F, t, r) with confidence intervals, effect sizes, degrees of freedom and P value noted<br>*Give P values as exact values whenever suitable.*
☒	☐	For Bayesian analysis, information on the choice of priors and Markov chain Monte Carlo settings
☒	☐	For hierarchical and complex designs, identification of the appropriate level for tests and full reporting of outcomes
☐	☒	Estimates of effect sizes (e.g. Cohen's *d*, Pearson's *r*), indicating how they were calculated

Our web collection on statistics for biologists contains articles on many of the points above.

Software and code

Policy information about availability of computer code

Data collection	In-house software from Olink was used to normalise protein expression data.
Data analysis	Custom code was used to conduct data analyses, which are made available at https://github.com/robertfhillary/vqtls-uk-biobank. The DOI is https://doi.org/10.5281/zenodo.11246859. The OSCA software (version 0.46) was used to conduct vQTL analyses. PLINK (version 1.9) was used to prepare outputs from genetic models and genetic data. VEP (version 110) and WGSA (version 0.95) were used for variant annotation, in addition to ANNOVAR (no version info).

For manuscripts utilizing custom algorithms or software that are central to the research but not yet described in published literature, software must be made available to editors and reviewers. We strongly encourage code deposition in a community repository (e.g. GitHub). See the Nature Portfolio guidelines for submitting code & software for further information.

Data

Policy information about availability of data

All manuscripts must include a data availability statement. This statement should provide the following information, where applicable:

- Accession codes, unique identifiers, or web links for publicly available datasets
- A description of any restrictions on data availability
- For clinical datasets or third party data, please ensure that the statement adheres to our policy

The genome-wide vQTL and GEI summary statistics generated in this study have been deposited in the Synapse database under the DOI: https://doi.org/10.7303/syn61514369. The underlying NPX measures are available through the UK Biobank Research Analysis Portal (https://www.ukbiobank.ac.uk/enable-your-research).

Research involving human participants, their data, or biological material

Policy information about studies with human participants or human data. See also policy information about sex, gender (identity/presentation), and sexual orientation and race, ethnicity and racism.

Reporting on sex and gender	The UK Biobank collected information on biological sex at baseline (field ID 31). Therefore, we report sex as a baseline characteristic in our study. We also conduct sex-stratified association tests as additional analyses because we identified differential associations between protein biomarkers and health-related outcomes in males and females. This is justified in the text given that we outline examples where opposing associations in males and females would cancel each other out in the whole UKB-PPP sample.
Reporting on race, ethnicity, or other socially relevant groupings	Our sample was separated into discovery (n=34,557) and replication subsets (n=17,806). The discovery set included participants who were of self-reported European ancestry. The remaining samples comprised the replication set and included 10,840 White, 931 African, 920 Central/South Asian, 308 Middle Eastern, 262 East Asian, and 97 admixed American individuals (as defined by data field 21000). We have outlined clearly that ethnicity was self-reported in our study.
Population characteristics	Summary data for all covariates and their associations with protein levels are shown in Supplementary Data 21-23.
Recruitment	500,000 UK Biobank participants were assessed between 2006 and 2010 in 22 assessment centres throughout the UK, covering a variety of different settings to provide socioeconomic and ethnic heterogeneity and urban–rural mix. This ensured a broad distribution across all exposures to allow the reliable detection of generalisable associations between baseline characteristics and health outcomes. However, it remains likely that self-enrollment into the study has resulted in selection bias within our sample. This is highlighted as a limitation in our Discussion.
Ethics oversight	All participants provided informed consent. This research has been conducted using the UK Biobank Resource under approved application numbers 65851, 20361, 26041, 44257, 53639, 69804.

Note that full information on the approval of the study protocol must also be provided in the manuscript.

Field-specific reporting

Please select the one below that is the best fit for your research. If you are not sure, read the appropriate sections before making your selection.

☒ Life sciences ☐ Behavioural & social sciences ☐ Ecological, evolutionary & environmental sciences

For a reference copy of the document with all sections, see nature.com/documents/nr-reporting-summary-flat.pdf

Life sciences study design

All studies must disclose on these points even when the disclosure is negative.

Sample size	No sample size calculations were performed. The justification for the sample size is that it is the largest available sample for the objectives of the study. This study included a subset of the UK Biobank study (UKB-PPP sample). The sample includes 54,219 participants and consists of (i) a randomised subset of 46,595 UKB participants at the baseline visit, (ii) 6,376 individuals at the baseline selected by the UKB-PPP consortium members and (iii) 1,268 individuals who participated in the COVID-19 repeat imaging study. This represents, to our knowledge, the largest available combined sample of genomics and proteomics, which allowed us to undertake our vQTL and GEI tests.
Data exclusions	No exclusions were conducted.<br>Inclusion strategy is outlined above.
Replication	Replication was successful. The UKB-PPP sample was separated into discovery (n=34,557) and replication subsets (n=17,806). The discovery set included participants who were of European ancestry and present in Olink measurement batches 1-6. The remaining samples comprised the replication set.
Randomization	Individuals were randomised across eight experimental batches for Olink protein measurements. In total, 46,595/54,219 UKB-PPP samples were randomly selected from baseline. The remaining samples were pre-selected by consortium members.

Blinding

Blinding was not relevant to our study. Individuals were randomly assigned to measurement batches, after which there was no group allocation relevant to the study (i.e. cases and controls). Therefore, blinding was not performed.

Reporting for specific materials, systems and methods

We require information from authors about some types of materials, experimental systems and methods used in many studies. Here, indicate whether each material, system or method listed is relevant to your study. If you are not sure if a list item applies to your research, read the appropriate section before selecting a response.

Materials & experimental systems

n/a	Involved in the study
☒	☐ Antibodies
☒	☐ Eukaryotic cell lines
☒	☐ Palaeontology and archaeology
☒	☐ Animals and other organisms
☒	☐ Clinical data
☒	☐ Dual use research of concern
☒	☐ Plants

Methods

n/a	Involved in the study
☒	☐ ChIP-seq
☒	☐ Flow cytometry
☒	☐ MRI-based neuroimaging

Plants

Seed stocks

Report on the source of all seed stocks or other plant material used. If applicable, state the seed stock centre and catalogue number. If plant specimens were collected from the field, describe the collection location, date and sampling procedures.

Novel plant genotypes

Describe the methods by which all novel plant genotypes were produced. This includes those generated by transgenic approaches, gene editing, chemical/radiation-based mutagenesis and hybridization. For transgenic lines, describe the transformation method, the number of independent lines analyzed and the generation upon which experiments were performed. For gene-edited lines, describe the editor used, the endogenous sequence targeted for editing, the targeting guide RNA sequence (if applicable) and how the editor was applied.

Authentication

Describe any authentication procedures for each seed stock used or novel genotype generated. Describe any experiments used to assess the effect of a mutation and, where applicable, how potential secondary effects (e.g. second site T-DNA insertions, mosiacism, off-target gene editing) were examined.
